# Establishment of a Blocking ELISA Detection Method for Against African Swine Fever Virus p30 Antibody

**DOI:** 10.3389/fvets.2021.781373

**Published:** 2021-12-17

**Authors:** Xuexiang Yu, Xianjing Zhu, Xiaoyu Chen, Dongfan Li, Qian Xu, Lun Yao, Qi Sun, Ahmed H. Ghonaim, Xugang Ku, Shengxian Fan, Hanchun Yang, Qigai He

**Affiliations:** ^1^College of Veterinary Medicine, Huazhong Agricultural University, Wuhan, China; ^2^State Key Laboratory of Agricultural Microbiology, Wuhan, China; ^3^The Cooperative Innovation Center for Sustainable Pig Production, Wuhan, China; ^4^Desert Research Center, Cairo, Egypt; ^5^College of Veterinary Medicine, China Agricultural University, Beijing, China

**Keywords:** African swine fever virus, blocking ELISA, diagnosis, monoclonal antibodies, p30

## Abstract

African swine fever (ASF) is a highly lethal hemorrhagic viral disease of domestic pigs caused by African swine fever virus (ASFV). A sensitive and reliable serological diagnostic assay is required, so laboratories can effectively and quickly detect ASFV infection. The p30 protein is abundantly expressed early in cells and has excellent antigenicity. Therefore, this study aimed to produce and characterize p30 monoclonal antibodies with an ultimate goal of developing a monoclonal antibody-based enzyme-linked immunosorbent assay (ELISA) for ASFV antibody detection. Three monoclonal antibodies against p30 protein that were expressed in *E. coli* were generated, and their characterizations were investigated. Furthermore, a blocking ELISA based on a monoclonal antibody was developed. To evaluate the performance of the assay, 186 sera samples (88 negative and 98 positive samples) were analyzed and a receiver-operating characteristic (ROC) analysis was applied to determine the cutoff value. Based on the ROC analysis, the area under the curve (AUC) was 0.997 (95% confidence interval: 99.2 to 100%). Besides, a diagnostic sensitivity of 97.96% (95% confidence interval: 92.82 to 99.75%) and a specificity of 98.96% (95% confidence interval: 93.83 to 99.97%) were achieved when the cutoff value was set to 38.38%. Moreover, the coefficients of inter- and intra-batches were <10%, indicating the good repeatability of the method. The maximum dilution of positive standard serum detected by this ELISA method was 1:512. The blocking ELISA was able to detect seroconversion in two out of five pigs at 10 Dpi and the p30 response increasing trend through the time course of the study (0–20 Dpi). In conclusion, the p30 mAb-based blocking ELISA developed in this study demonstrated a high repeatability with maximized diagnostic sensitivity and specificity. The assay could be a useful tool for field surveillance and epidemiological studies in swine herd.

## Introduction

African swine fever (ASF), caused by African swine fever virus (ASFV), is a highly contagious hemorrhage lethal disease of domestic and wild pigs and is responsible for serious economical losses, international trading, and adverse sociophysical impacts (1–6). It is causing a serious deterioration and incalculable economic impact due to its fast spread. The disease was first reported in Kenya in the 1910s, and 51 countries are currently affected by African Swine Fever (OIE) (7–10). ASFV is a large and complex double-stranded DNA virus with icosahedral morphology (5, 6, 11). Although it was generally considered that there is only one serotype of ASF virus, the classification of ASFV isolates in eight different serogroups based on a hemadsorption inhibition assay (HAI) (12). However, genetic characterization of all the ASF virus isolates known so far has demonstrated 24 geographically related genotypes with numerous subgroups (1, 7, 10). ASFV was first reported in China in August 2018; analysis showed that the causative strain belonged to the p72 genotype II and CD2v serogroup 8 (13, 14).

Due to the presence of seropositive animals to subacute or chronic forms of ASF, there is always a need for an accurate serological diagnosis. Serological assays are the most commonly used diagnostic tests due to their simplicity, comparatively low cost, and their necessitating few specialized pieces of apparatus or facilities. Since there is no vaccine against ASF, the presence of ASFV antibodies always indicates current or historic infection (7, 15). Also, 2–10% of animals recover from the acute form may act as persistent viral shedding sources (7). Studies have shown that the infectious virus genome was detected in tissues (retropharyngeal and submandibular lymph nodes, bone marrow, and tonsil) but was not detected in whole blood from the recovered animals (16). In addition, there were reported variant strains in China, with relatively weak virulence and atypical clinical symptoms (17, 18). Since the antibody IgG appears 7–10 days post-infection and persists for months and even lifetime (7, 15). Therefore, a sensitive and reliable serological diagnostic assay is required, so laboratories can effectively and quickly detect ASFV infection. Corresponding to this, identifying potential antigenic ASFV protein targets that suit to develop a diagnostic assay is very important, of which the p30, p72, and p54 are the best targets (19–25).

The major capsid protein p72 is used to establish numerous ELISA-based serological assays (24, 25). Among them, the p72 protein is mostly used in research. It has good immunogenicity, strong conservation, and high expression. The blocking-ELISA for ASFV antibody detection depends on the use of monoclonal antibodies against p72 (Ingenasa-Ingezim PPA COMPAC K3; Ingenasa, Madrid, Spain) (26, 27), but the detection time upon using p30 protein as the antigen can be earlier than that of p72 protein (20). The p54 protein in different regions has a certain variation in the amino acid sequence, which is easy to cause false-negative results, so it is usually not used as a detection antigen for ASF (20). Compared with the p54 and p72 proteins, the p30 protein is produced earlier and can neutralize the virus before or after the virus adsorption to the cell. The p30 protein is abundantly expressed early in cells and has excellent antigenicity (20); it is also an important target for early diagnosis of the virus (28–30). Therefore, p30 protein can be used as an antigen to develop the early detection antibody method of ASFV infection.

In the current study, monoclonal antibodies (mAbs) against recombinant protein p30 were generated and their characterizations were investigated. Due to the high specificity of blocking ELISA, a blocking ELISA based on p30 mAb was developed. The established blocking ELISA showed high diagnostic sensitivity and specificity for ASFV antibody detection, providing a new tool for ASFV antibody detection.

## Materials and Methods

### Production of Recombinant p30 in *Escherichia coli*

The ASFV CP204L (582 bp) gene sequence from positive samples during the surveillance was used for the preparation of p30 recombinant protein fragments. His-tagged full-length CP204L constructs were cloned into the pET-30a vector, and recombinant proteins were expressed in *E. coli*, as described previously (31, 32). CP204L was amplified by PCR using a forward primer 5′-GGCCATGGCTATGGATTTTATTTTAAATAT-3′ and a reverse primer 5′-CCGCTCGAGTTTTTTTTTTAAAAGTTTA-3′. The primers were designed based on African swine fever virus isolate Pig/HLJ/2018 (accession. no. MK333180.1) (16, 17). The single underline is the sequence of the restriction sites of *Nco*I and *Xho*I. Briefly, polymerase chain reaction (PCR) amplified CP204 gene (582 bp) and pET-30a vector were digested with *Nco*I and *Xho*I (TakaRa, TakaRa Biotechnology Co., Ltd., Dalian, China) restriction enzymes and accordingly ligated with T4 DNA ligase. Recombinant genes were then transformed to Transetta (DE3) *E. coli* competent cells (TransGen Biotech Co., Ltd., Beijing, China) and incubated overnight at 37°C in an agar plate containing kanamycin. Subsequently, perfection of the correct insert was checked by PCR and positive samples were confirmed by DNA sequencing (Sangon Biotech Co., Ltd., Shanghai, China).

### Expression and Purification of Recombinant ASFV-P30 Protein

Expression of the p30 protein was facilitated by adding 1 mM isopropyl-β-D-1-thiogalactoside (IPTG), and successful expression was examined by sodium dodecyl sulfate-polyacrylamide gel electrophoresis (SDS-PAGE) analysis of cell lysates. To purify p30 recombinant protein, bacterial cells were harvested by centrifugation, resuspended in pre-cold PBS (50 ml/liter of bacterial culture) (Dalian Meilun Biotechnology Co., Ltd., Dalian, China), and lysed by high-pressure crushing. After centrifugation at 12,000 rpm for 30 min, supernatants were collected and filtered through a 0.22-μm filter and purified using a Ni-NTA resin-based column. The protein sample p30 was taken for analysis by SDS-PAGE; anti-His mAb (Proteintech Group, Inc., Rosemont, IL, USA) and ASFV-positive serum were used as primary antibodies for Western blot verification.

### mAb Production

As previously described (33, 34), 4–6-week-old BALB/C mice were immunized with 100 μg/mouse of purified p30 protein mixed with an equal volume of incomplete Freund's adjuvant (Sigma-Aldrich (Shanghai) Trading Co. Ltd., Shanghai, China). Mice were immunized intraperitoneally three times with 2 weeks between each immunization. The mice were euthanized 3 days after the final immunization, after which splenocytes were collected and fused with SP2/0 myeloma cells. After fusion, cells were cultured in 96-well plates (Corning Incorporated Co., Ltd., Kennebunk, ME, USA) in HAT selection media. Cell supernatants were assayed 10 days post cell fusion, and wells with confluent hybridomas were initially screened by indirect ELISA using p30 recombinant protein as a coating antigen. Then, the positive culture supernatants were screened for p30-specific antibodies by immunofluorescence assay (IFA) on PMA cells infected with ASFV which was isolation during the surveillance. Hybridoma clones that produced p30-specific antibodies were subcloned into single-cell clones (monoclones).

### Indirect ELISA

Purified recombinant p30 protein constructs were coated on flat-bottom polystyrene plates (1 μg/ml; 100 μl/well) in carbonated coating buffer (pH 9.6) and incubated overnight at 4°C. The plate was washed five times with PBST (0.05% Tween in PBS, v/v), and the plate was blocked with 5% skimmed milk in PBS, for 1 h at 37 °C. After washing the plates as above, 50 μl undiluted hybridoma supernatants was added. Positive serum from mice immunized with p54 recombinant protein and negative serum from unimmunized mice, diluted 1:10,000, were also included in duplicate as a control. The plate was incubated for 30 min at 37°C, and a washing step was repeated. Thereafter, horseradish peroxidase (HRP) conjugated goat anti-mouse IgG (Proteintech Group, Inc., Rosemont, IL, USA) diluted 1:10,000 was added and incubated for 30 min at 37°C. Following washing five times, reaction was developed by adding a chromogenic substrate solution (TMB) (Beyotime Biotechnology Co., Ltd., Shanghai, China) for 10 min and stopped with Stop Solution for TMB Substrate (Beyotime Biotechnology Co., Ltd., Shanghai, China). The plates were read at 630 nm.

### IFA

IFA tests on ASFV-infected cells were conducted on porcine alveolar macrophage (PAM) cells infected with ASFV (The virus was isolated and produced by PAM cells, and the virus TCID_50_ was measured by the Reed–Muench method. The virus was stored at −80°C. The virus was isolated and stored in the Animal Biosafety Level 3 Laboratory of Huazhong Agricultural University.). PAM cells were collected from 20 to 30-day-old pigs, and the cells were plated on 96-well plates in 10% FBS (Gibco, Thermo Scientific, USA) RPMI 1640 medium (Gibco, Thermo Scientific, Waltham, MA, USA) at 37°C with 5% CO_2_ and infected with ASFV at an MOI of 0.1. At 36 hpi, cell monolayers were fixed with 4% paraformaldehyde in PBS for 30 min at room temperature. The above operations are carried out in the Animal Biosafety Level 3 Laboratory of Huazhong Agricultural University. Cells were incubated with anti-p30 mAb followed by incubation with FITC conjugated goat anti-mouse IgG (ABclonal Technology Co., Ltd., Wuhan, China). Nuclei were stained with DAPI, and the plates were examined using the fluorescence microscope (EVOS FL Auto, Thermo Fisher Scientific, USA).

### Serum Standard and Testing Samples

The serum samples were used for blocking ELISA development and validation. One hundred and eighty-six serum samples were analyzed with the established blocking ELISA, including 88 negative sera and 98 ASFV-positive sera. These 88 samples were collected before the outbreak of ASFV in China and were confirmed to be negative by the commercial ASFV antibody detection kit (INgezim PPA COMPAC, Ingenasa, Madrid, Spain). All the 98 ASFV-positive samples used in this study were from clinically infected pigs, and their positivity was determined by the commercial ASFV antibody detection kit (INgezim PPA COMPAC, Ingenasa, Madrid, Spain). ASFV-positive and -negative sera were kindly gifted by the National African Swine Fever Reference Laboratory of the China Animal Health and Epidemiology Center.

### Procedure for ASFV Indirect ELISA and Blocking ELISA

The purified p30 mAb was labeled with horseradish peroxidase (HRP) (Shandong Galaxy Bio-Tech Co., Ltd., Jining, China) to establish a blocking ELISA antibody detection method. Purified recombinant p30 protein constructs were coated on flat-bottom polystyrene plates (0.5 μg/ml; 100 μl/well) in carbonated coating buffer (pH 9.6) and incubated overnight at 4°C. The plate was washed five times with PBST (0.05% Tween in PBS, v/v), and the plate was blocked with 2% skimmed milk in PBS, for 1 h at 37°C. After washing, 100 μl of the diluted control and testing sera was added and incubated at 37°C for 30 min, and a washing step was repeated. All control and testing sera samples were diluted 1:1 in dilution buffer (0.01% Tween 20 in 1× PBS). Next, 100 μl of biotinylated anti-p30 mAb (HPR-anti-p30 mAb; 1 μg/ml) was added into each well, and the plate was incubated at 37°C for another 30 min. Following extensive washing, reaction was developed by adding chromogenic substrate solution (TMB) (Beyotime Biotechnology Co., Ltd., Shanghai, China) for 10 min and stopped with Stop Solution for TMB Substrate (Beyotime Biotechnology Co., Ltd., Shanghai, China). The plates were read at 630 nm, and the raw data were transformed to an Excel sheet and consequently the percent of inhibition (PI value) of each test sample was calculated using the formula: PI (%) = [(OD_630_ value of negative controls – OD_630_ value of sample)/OD_630_ value of negative controls] ×100%, as described by Wang et al. (35).

### Cut-Off Value, Diagnostic Sensitivity, and Specificity Determination

To calculate the optimal cutoff value, and associated diagnostic sensitivity and specificity, serum samples from individual pigs of known ASFV-positive and -negative testing sample were tested by blocking ELISA. Receiver operating characteristic (ROC) analysis and degree of agreement (kappa value) were analyzed using SPSS software for windows, version 26.0 (IBM, Armonk, NY, USA). Using the commercial blocking ELISA kit as a standard evaluating method, the sensitivity and specificity of the established ELISA were calculated by the web-based MedCalc statistical software [https://www.medcalc.org/calc/diagnostic.test.php (accessed on 7 June 2021)].

### Assessment of Blocking ELISA Specificity and Repeatability

To confirm the specificity, the developed blocking ELISA was used to detect six polyclonal anti-sera against other swine viruses (PCV2, PCV3, CSFV, PRV, PRRSV, O-FMDV).

The repeatability of blocking ELISA was assessed by running 10 control sera (three positive control, three medium-positive control, and four negative control). The within-run assay precision was calculated using a standard serum tested on three plates in one run, and the between-run precision was calculated from a standard serum tested in three different runs. Means, standard deviations, and percent coefficient of variation (% *CV*) were calculated using SPSS software for windows, version 26.0.

### Detection Antibody in ASFV-Infected Pig Sera, ASFV Positive Standard Serum

ASFV-infected pig sera were collected at different time-points (0, 5, 10, 15, and 20 Dpi) from experimentally infected pigs. Five sera were collected at each stage. The ASFV-infected pig sera were donated by Harbin Veterinary Research Institute. The ASFV-positive standard serum (no. 202101) and the ASFV-positive standard serum against CD2v-negative (no. 202101) (swine sera infected with ASFV delete the CD2v gene) were purchased from the China Veterinary Drug Administration. The ASFV-positive standard serum and the ASFV-positive standard serum against CD2v-negative at different dilutions were titrated with twofold dilutions from 1:4 to 1:1,024. All collected serum samples were tested by the p30 mAb-based blocking ELISA.

## Results

### Antigen Preparation

**Figure 1:**
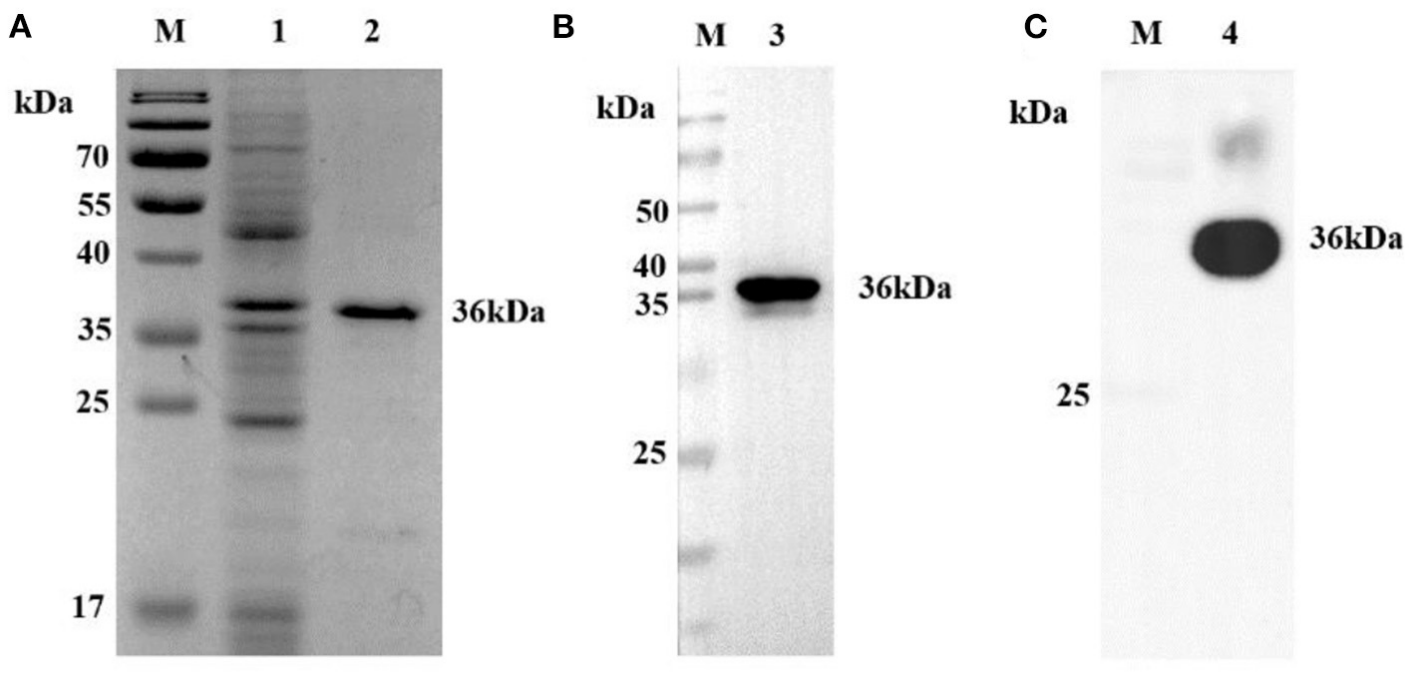
Analysis of p30 protein. **(A)** SDS-PAGE analysis of recombinant p30 protein. The recombinant protein can be seen at 36 kD (Black). **(B)** Western blot analysis of recombinant p30 protein with anti-His tag antibody. The recombinant protein can be seen at 36 kD (Black). It can be seen that the recombinant protein can react specifically with anti-His mAb. **(C)** Western blot analysis of recombinant p30 protein with ASFV positive serum. The recombinant protein can be seen at 36 kD (Black). It can be seen that the recombinant protein can react specifically with ASFV positive serum; M: protein Marker; 1: Negative control; 2, 3, 4: p30 protein.

The synthetic DNA fragment of the CP204L gene from ASFV-positive DNA was cloned and expressed in *E. coli* as a His-tagged recombinant protein. The p30 protein was expressed at a high level but formed inclusion bodies. Coomassie blue staining showed a sharp band at the predicted size of the purity His-tagged p30 (~36 kDa) in sodium dodecyl sulfate–polyacrylamide gel electrophoresis (SDS-PAGE) (Figure 1A). The identity of the recombinant protein was further confirmed by Western blot analysis using an anti-His mAb (Figure 1B) and the ASFV-positive serum (Figure 1C).

### Generation of MAbs Against ASFV p30

**Figure 2:**
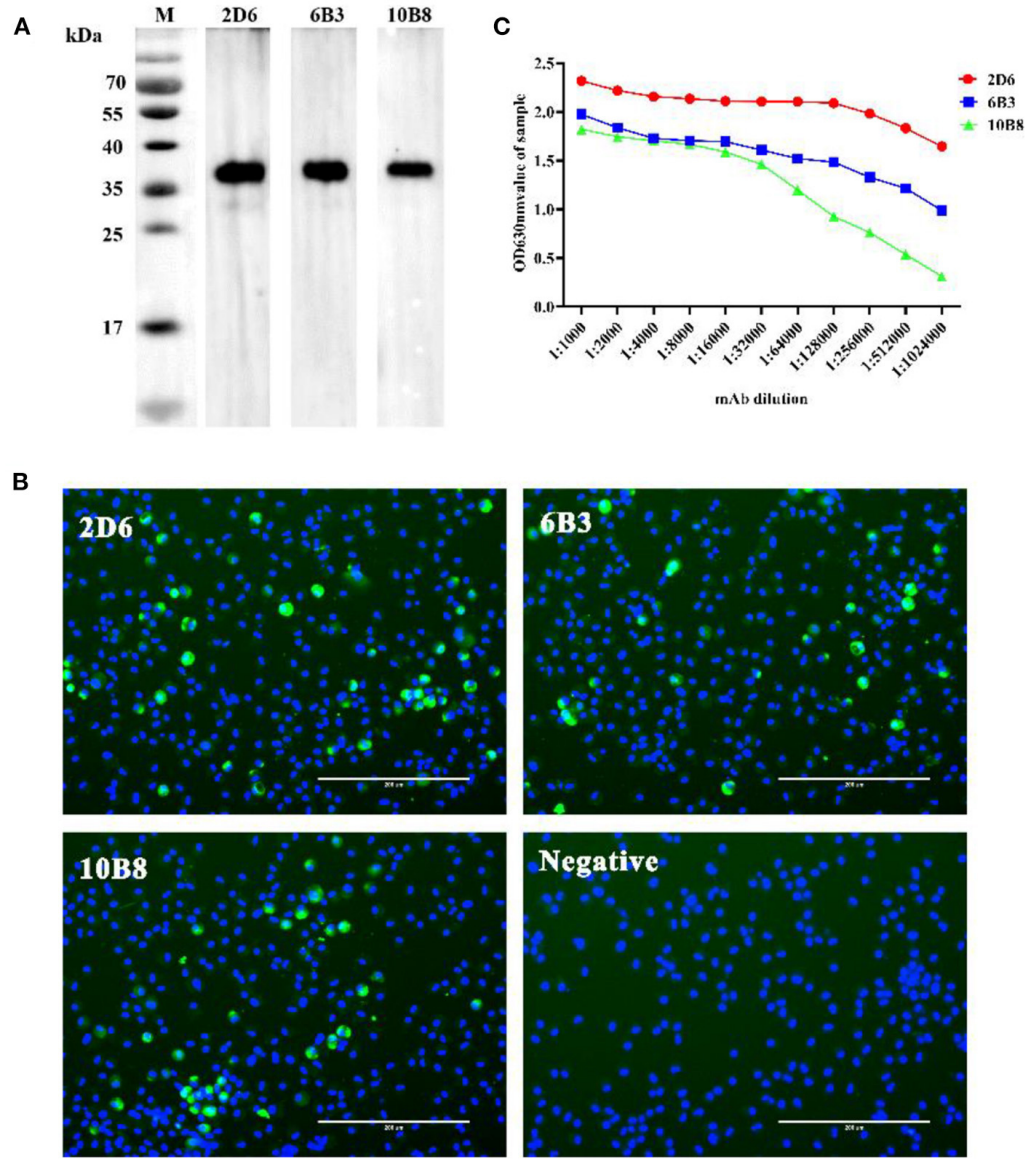
Selection of p30-specific mAb for use in blocking ELISA. **(A)** Western blot analysis anti-p30 mAbs. The three anti-p30 monoclonal antibodies can react specifically with recombinant protein at 36 kD (Black). **(B)** IFA performed on PAMs that were infected with ASFV. Cells were incubated with p30-specific mAbs listed on the top of each panel and stained with FITC-conjugated goat anti-mouse IgG (Green). Cell nucleus was counterstained with DAPI (Blue). Scale bars, 200 μm. **(C)** Different mAbs titer test results. The OD value of mAb-2D6 (Red) at any dilution is higher than mAb-6B3 (Blue) and mAb-10B8 (Green). It is shown that mAb-2D6 has the highest antibody titer.

To generate anti-p30 mAbs, mice were immunized with recombinant p30 protein. After the fusion process, supernatants from the resulting hybridoma cells were screened by p30 indirect ELISA, Western blot analysis (Figure 2A), and IFA using PAMs infected with ASFV (Figure 2B). One mAb from each primary clone, mAb 2D6, 6B3, and 10B8, was selected for further characterization. The different mAbs at different dilutions were titrated with 2-fold dilutions from 1:1,000 to 1:1,024,000 (Figure 2C). Furthermore, isotypes of mAbs were characterized using the mouse Ig isotyping kit (Southern Biotechnology Associates, Inc., Birmingham, USA) and all were found to be IgG1 with kappa light chain (Table 1).

**Table 1:** Identification of subclasses of p30 monoclonal antibodies.

	**Monoclonal antibodies**		
	**2D6**	**6B3**	**10B8**
Ig subclass	IgG1	IgG1	IgG1
Light chain type	κ	κ	κ

### Assessing Potential Uses of p30 Monoclonal Antibodies for Blocking ELISA

**Figure 3:**
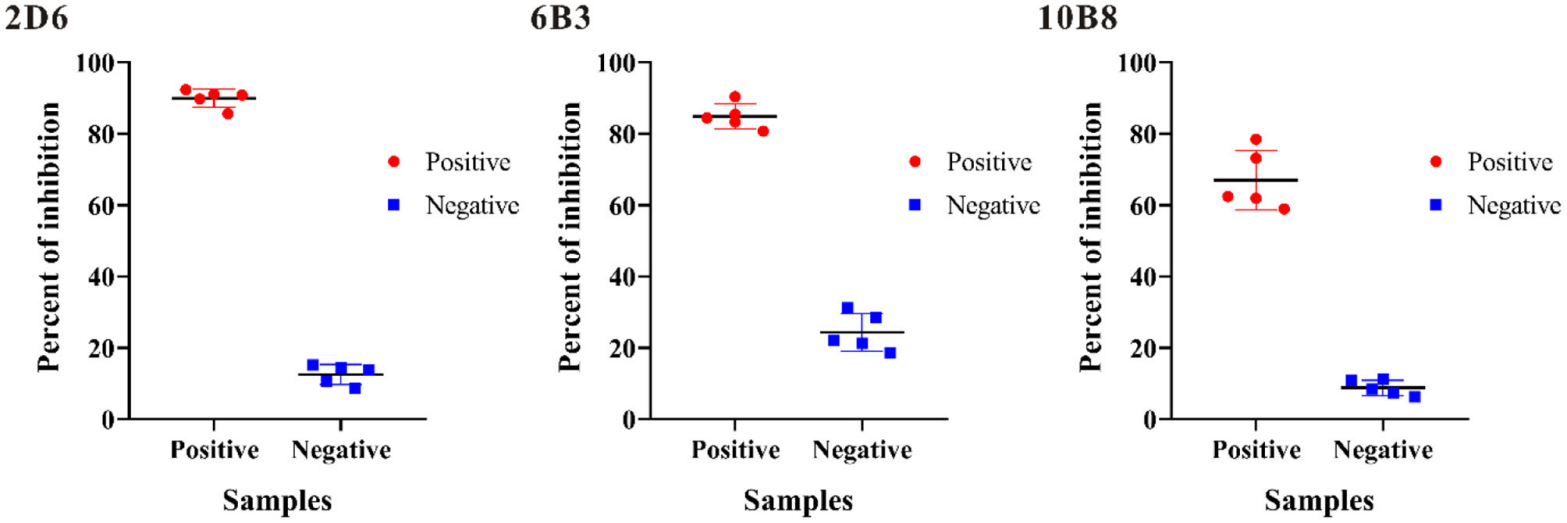
Investigation of p30 monoclonal antibodies on blocking ELISA for ASF detection. The percent of inhibition of five positive samples (Red) and five negative samples (Blue) were determined, and the average percent of inhibition of negative and positive samples was displayed.

To evaluate the potential use of these anti-p30 monoclonal antibodies as a diagnostic reagent for ASFV antibody detection, blocking ELISA based on each monoclonal antibody was investigated. Five positive sera and five negative sera were selected to determine which p30 monoclonal antibody will have a good performance to be applied in blocking ELISA. Each sample was tested with the blocking ELISA at a dilution of 1:2, and the percent of inhibition (PI value) of each sample was calculated (Figure 3). The result revealed that all test-positive samples were able to block mAb-2D6 by greater than others.

### Standardization and Determining the Negative Cut-Off Value for Blocking ELISA

**Figure 4:**
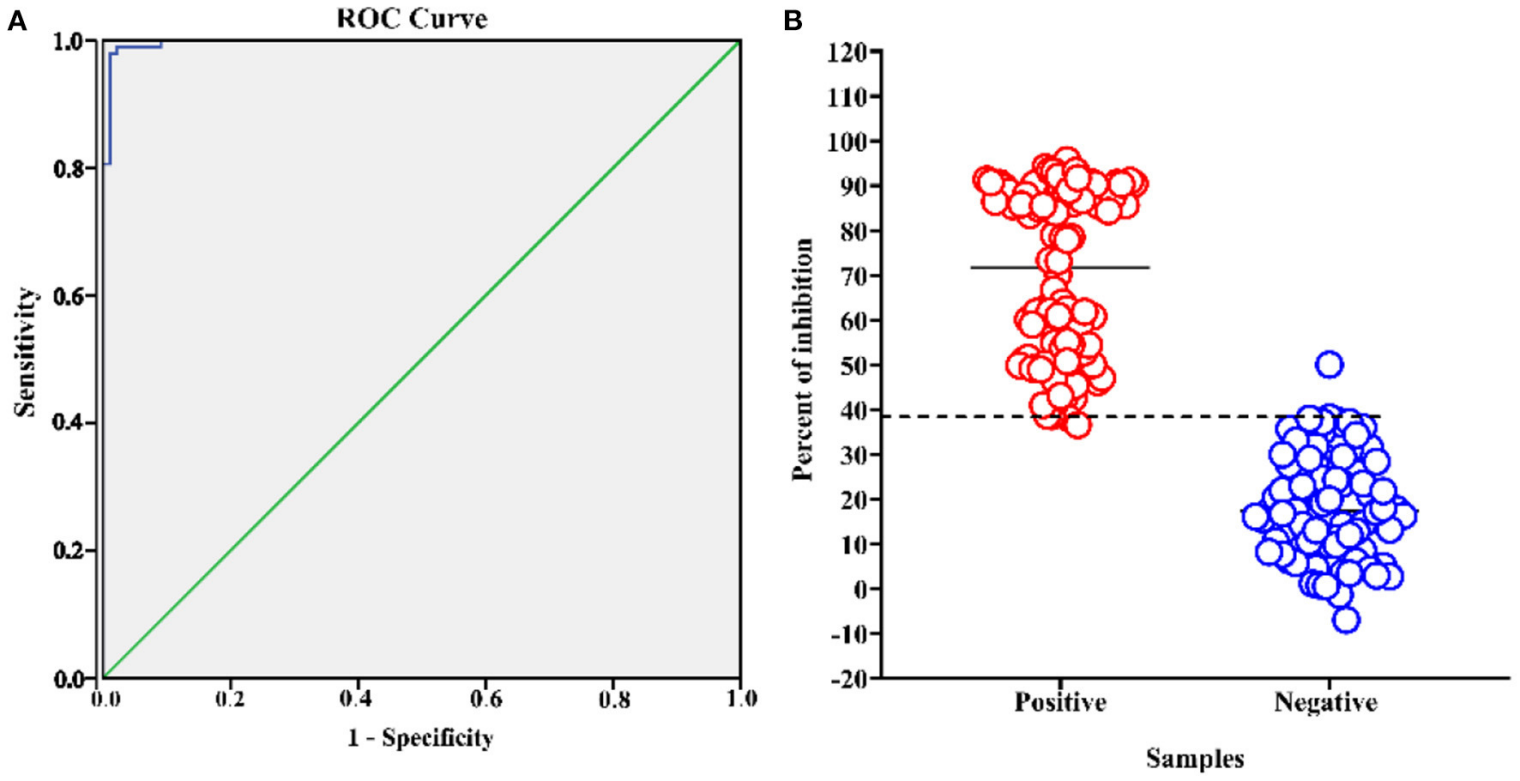
ASFV p30-based blocking ELISA analysis of serum samples. The analysis was performed on known ASFV-negative samples (*n* = 88) and known ASFV-positive samples (*n* = 98). **(A)** ROC analysis of blocking ELISA results while the area under the curve (AUC) of the test was 0.997. **(B)** Interactive dot plot diagram showing the blocking value of serum samples while the cut-off value was set to 38.38%.

After optimizing the protocol for competitive ELISA, a total of 186 pig serum samples (88 negative samples and 98 positive samples) were tested to assess the performance of the assay. These samples were classified as ASFV seronegative or ASFV seropositive according to their known origin and using a commercial ASFV antibody detection kit (INgezim PPA COMPAC, Ingenasa, Madrid, Spain). All samples were tested in duplicate by the established blocking ELISA, and the percent of inhibition value of each sample was calculated. An ROC curve statistical analysis was performed and allowed us to determine the cutoff value and estimate the diagnostic sensitivity and specificity of the assay (Figure 4A). In addition, an interactive dot plot diagram outlined the blocking value of these samples, as shown in Figure 4B. An AUC of 1 represents a perfect test, and an AUC above 0.9 indicates high accuracy of the assay. Based on the ROC analysis, the area under the curve (AUC) of the established test was 0.997 (95% confidence interval: 99.2 to 100%). Besides, a diagnostic sensitivity of 97.96% (95% confidence interval: 92.82 to 99.75%) and a specificity of 98.96% (95% confidence interval: 93.83 to 99.97%) were achieved when the cutoff value was set to 38.38%, demonstrating the high accuracy of the assay.

### Assessment of Blocking ELISA Specificity and Repeatability

To confirm the specificity, the developed blocking ELISA was used to detect six polyclonal anti-sera against other swine viruses (PCV2, PCV3, CSF, PR, PRRSV, O-FMDV). All sera yielded a negative result in the blocking ELISA with a blocking value much lower than the cutoff value. Thus, non-specific positive swine sera were clearly discriminated from the ASFV-positive sera, suggesting that the established blocking ELISA has a satisfactory analytical specificity.

Reproducibility determines whether an entire experiment or study can be reproduced. In this study, 12 serum samples (eight positive samples and four negative samples) were selected for testing by the developed blocking ELISA while the intra- and inter-assay variations were determined by calculating the coefficient of variation (*CV*%). The coefficient of variation (*CV*) <10% was considered to have an adequate repeatability. In this study, an intra-assay *CV* ranging from 1.09 to 8.56% and an inter-assay *CV* ranging from 1.21 to 9.92% were observed, indicating that the p30-based blocking ELISA is highly repeatable.

### Antibody Response to p30 in ASFV-Infected Pigs

**Figure 5:**
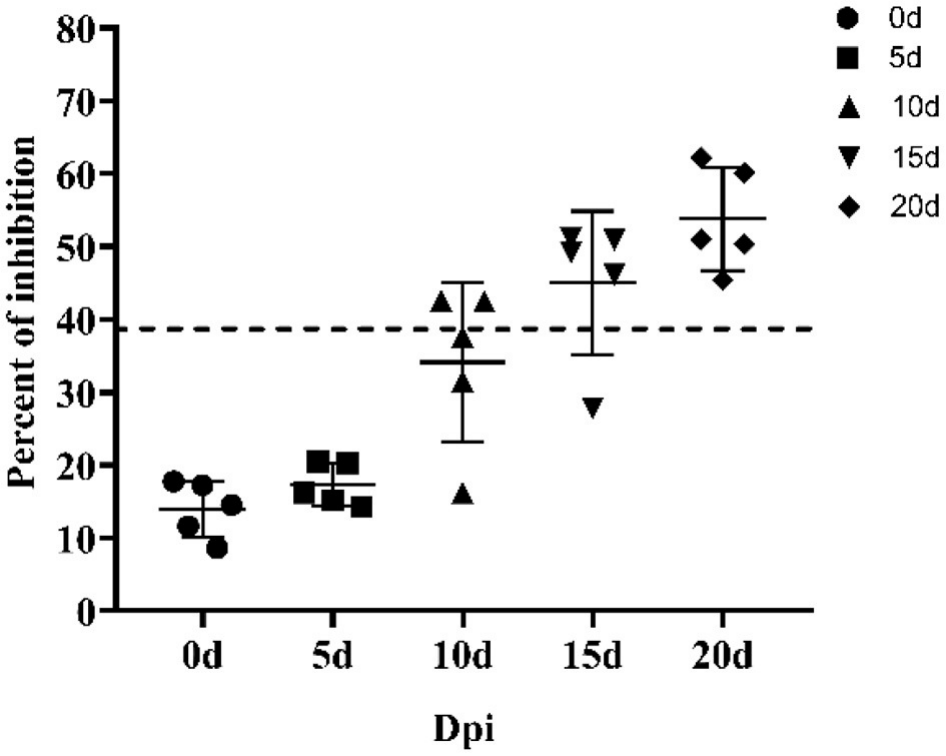
Kinetics of antibody response in serum from ASFV-infected pigs. Serum samples were collected from six pigs infected by ASFV at 0, 5, 10, 15, and 20 days post inoculation. The dashed line represents the cut-off of blocking ELISA.

Next, we applied the p30-based blocking ELISA to determine the humoral immune response in ASFV-infected pigs. The ASFV-specific antibody response was determined using blocking ELISA. As shown in Figure 5, the antibody response against p30 protein was detected seroconversion as early as 10 Dpi in two out of five pigs and the p30 response peaked around 20 Dpi.

### Analytical Sensitivity of the p30-Based Blocking ELISA

**Figure 6:**
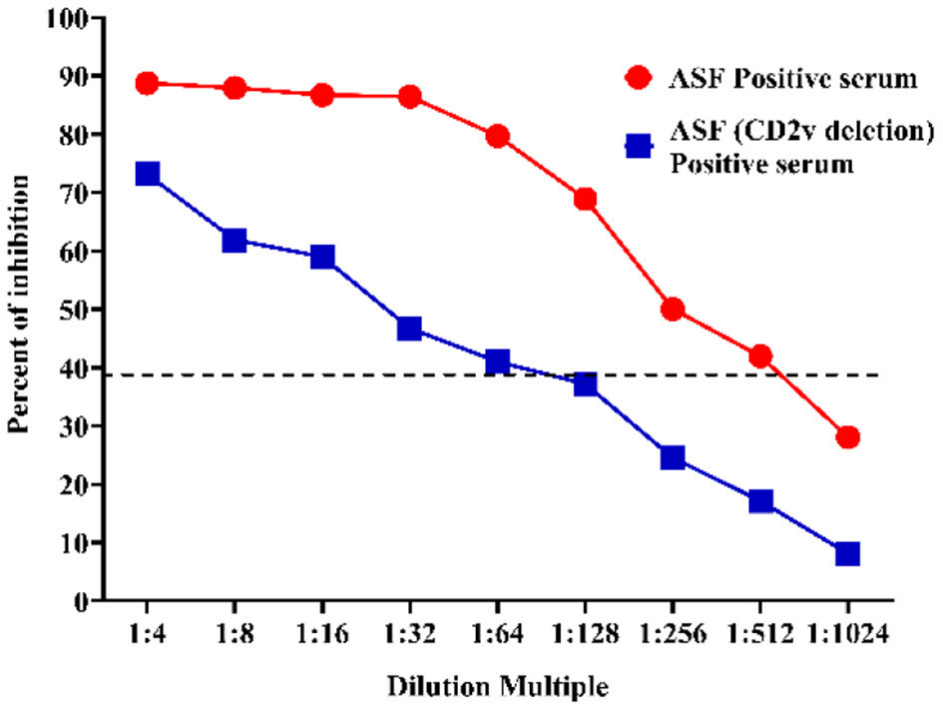
Sensitivity assay. The ASFV-positive standard serum (Red) and the ASFV-positive standard serum against CD2v-negative (Blue) at different dilutions were titrated with 2-fold dilutions from 1:4 to 1:1,024. The dashed line represents the cut-off of blocking ELISA.

After the assay conditions were optimized, the analytical sensitivity of the p30-based blocking ELISA was evaluated using the ASFV-positive standard serum and the ASFV-positive standard serum against the CD2v-negative one. The maximum dilution of ASFV-positive standard serum detected at different dilutions was 1:512, and the ASFV-positive standard serum against CD2v-negative maximum dilution was 1:64, indicating that the p30-based blocking ELISA is highly sensitive (Figure 6).

## Discussion

ASFV causes a serious deterioration and incalculable adverse economic impact around the world especially in China which has the largest pig industry (13, 16, 17). Currently, there is no vaccine or other treatments available for ASFV. The principal strategy for control remains early detection, quarantine, and depopulation of affected herds. Cost-effective detection strategies are needed for conducting high-throughput surveillance (2, 7, 24–27). Although the seroconversion time was later than the virus genome-detected time, no significant symptoms were found in the variant strain-infected animals, and the infected pigs underwent intermittent detoxification (18). In addition, in several areas (Africa and Europe), many pigs or wild boar survived infection and presented no clinical signs of ASFV at the time of samplings, without the presence of ASFV attenuated variants (36–38); thus, we need to accurately detect the antibody of the animal to be tested to facilitate the determination of the infection of the pigs. Therefore, a sensitive and reliable serological diagnostic assay is required, so as laboratories can effectively and quickly detect ASFV infection (18, 26).

ELISA is considered a common tool to carry out serological surveillance. Among these ELISA methods, two main types of ELISAs have been employed in antibody detections. One is indirect ELISA, where coated antigens capture specific antibodies in serum samples directly. The other is blocking or competitive ELISA, where virus-specific antibodies in samples react with antigens to block or compete with the binding of a mAb to the antigens. The specificity of iELISA is generally influenced by high background due to the non-specific reaction of serum antibodies to contaminant antigens in the tests (39). In this study, we established a blocking ELISA for the detection of antibodies against ASFV in pig serum. The ELISA is a rapid, economical, and sensitive diagnostic method for screening large numbers of sera for antibodies. Additionally, the specificity of this method is supposed to be even higher due to the usage of mAbs. Blocking ELISAs were widely used for a broad range of applications concerning serological diagnosis of various diseases in different animal species (40, 41).

The p30 mAb-based blocking ELISA demonstrated good diagnostic sensitivity of 97.96% and specificity of 98.96%. Based on the selected cutoff value of 38.38%, 1 out of 88 negative sera samples showed false-positive results, with a PI value of 50.01, while 2 out of the 98 positive serum samples showed false-negative results with PI values of 36.59 and 38.03%. It has been reported that intrinsic and external factors, such as autoantibodies, sample quality, and sample storage conditions, can affect the serologic testing (41). Physical and chemical parameters can also affect the test results in the laboratory, such as hemolysis and lipemia (42). In our study, of the two false-negative serum samples and the one false-positive sample, these three serum samples were confirmed as negative by IFA.

The p30 mAb-based blocking ELISA was further validated for detecting seroconversion and monitoring the dynamic of antibody response in experimental pigs infected with ASFV. The blocking ELISA was able to detect seroconversion in two out of five pigs at 10 dpi. It detected an increasing trend of antibody response against p30 protein through the time course of the study (0–20 dpi). The detection of seroconversion at 10 dpi was consistent with the findings in previous studies (23, 43–45), so this detection method can be used as an early detection kit. The maximum dilution of the ASFV-positive standard serum and the ASFV-positive standard serum against the CD2v-negative one at different dilutions were 1:512 and 1:64, respectively, indicating that the p30-based blocking ELISA was highly sensitivity and it can detection for variant strains.

Furthermore, with the emergence of domestic attenuated strains and atypical clinical symptoms, antibody detection methods can be used as an effective means to detect infections. The antibody detection methods can be used to screen ASF antigen–antibody double-negative pigs upon introducing pigs into farm. Due to its simplicity concerning the coating antigen production, easiness to perform, and low cost, the test will be a useful tool for field surveillance and epidemiological studies in swine herd. The non-invasive test for a complete epidemiological investigation in the field is very important, especially ASF. In subsequent studies, an attempt should be made to establish an antibody detection method for oral fluid.

## Conclusion

This study prepared three monoclonal antibodies against the structural p30 protein of ASFV, and their diagnostic application was investigated. The p30 mAb-based blocking ELISA developed in this study demonstrated a high repeatability with maximized diagnostic sensitivity and specificity in laboratory settings. Through the aforementioned experiments and analysis, we conclude that the newly developed mAb 2D6-based blocking ELISA method offers a promising approach for a rapid and convenient ASFV serodiagnosis. The assay could be a useful tool for field surveillance and epidemiological studies in swine herd.

## Data Availability Statement

The original contributions presented in the study are included in the article/supplementary material, further inquiries can be directed to the corresponding author/s.

## Ethics Statement

The animal study was reviewed and approved by the Ethics Committee of the Faculty of Veterinary Medicine of the Huazhong Agricultural University.

## Author Contributions

XY, XZ, XK, SF, HY, and QH contributed to the conception or design of the work and the acquisition of data. XC, DL, and QX completed the data analysis. LY, QS, and AG drafted the manuscript and revised it critically for important intellectual content. All authors have critically read and edited the manuscript.

## Funding

This project was funded by the Epidemiological characteristics and evolution of African swine fever virus and early detection technology (31941004) and the research and application of key technology against African Swine Fever (2019ABA089).

## Conflict of Interest

The authors declare that the research was conducted in the absence of any commercial or financial relationships that could be construed as a potential conflict of interest.

## Publisher's Note

All claims expressed in this article are solely those of the authors and do not necessarily represent those of their affiliated organizations, or those of the publisher, the editors and the reviewers. Any product that may be evaluated in this article, or claim that may be made by its manufacturer, is not guaranteed or endorsed by the publisher.

## References

[1] Torre A Bosch J Iglesias I Muñoz M Mur L Martínez-López B. Assessing the risk of African swine fever introduction into the European Union by Wild Boar. Transb Emerg Dis. (2015) 62:272–9. 10.1111/tbed.12129 23926953

[2] Zakaryan H Revilla Y. African swine fever virus: current state and future perspectives in vaccine and antiviral research. Vet Microbiol. (2016) 185:15–19. 10.1016/j.vetmic.2016.01.016 26931386

[3] Gaudreault NN Madden DW Wilson WC Trujillo JD Richt JA. African swine fever virus: an emerging DNA arbovirus. Front Vet Sci. (2020) 7:215. 10.3389/fvets.2020.00215 32478103 PMC7237725

[4] Nguyen T Xuan D Thi T Van T Thi B Yong J. Molecular profile of African swine fever virus (ASFV) circulating in Vietnam during 2019–2020 outbreaks. Arch Virol. (2021) 166:885–90. 10.1007/s00705-020-04936-5 33454861

[5] Wang N Zhao D Wang X Wang J Zhang Y Wang M. Architecture of African swine fever virus and implications for viral assembly. Science. (2019) 366:640–4. 10.1126/science.aaz1439 31624094

[6] Salas ML Andrés G. African swine fever virus morphogenesis. Virus Res. (2013) 173:29–41. 10.1016/j.virusres.2012.09.016 23059353

[7] Food Food and Agriculture Organization of Animal Health (FAO) Beltrán-Alcrudo D FAO Reference Centre INIA-CISA Kramer SA FAO. African Swine Fever: Detection and Diagnosis. FAO (2017).

[8] OIE. Global Situation of African Swine Fever. Report No. 47:2016–2020. Available online at: https://rr-asia.oie.int/en/projects/asf/situational-updates-of-asf-in-asia-and-the-pacific/ (accessed December 15, 2020).

[9] OIE. Global Situation of African Swine Fever. Report No. 64: February 05 to February 18, 2021. Available online at: https://www.oie.int/en/document/report_64_current_situation_of_asf/ (accessed March 24, 2020).

[10] OIE. Global Control of African Swine Fever: A GF-TADs Initiative (2020–2025). Available online at: https://www.oie.int/en/global-action-needed-now-to-halt-spread-of-deadly-pig-disease/ (accessed July 17, 2020).

[11] Arias M Jurado C Gallardo C Fernández-Pinero J Sánchez-Vizcaíno JM. Gaps in African swine fever: analysis and priorities. Transb Emerg Dis. (2018) 65:235–47. 10.1111/tbed.12695 28941208

[12] Malogolovkin A Burmakina G Titov I Sereda A Gogin A Baryshnikova E. Comparative analysis of African swine fever virus genotypes and serogroups. Emerg Infect Dis. (2015) 21:312–5. 10.3201/eid2102.140649 25625574 PMC4313636

[13] Shengqiang G Jinming L Xiaoxu F Fuxiao L Lin L Qinghua W. Molecular characterization of African swine fever virus, China, 2018. Emerg Infect Dis. (2018) 24:2131–3. 10.3201/eid2411.181274 30141772 PMC6199985

[14] Sánchez-Vizcaíno J Mur L Gomez-Villamandos J Carrasco L. An update on the epidemiology and pathology of African swine fever. J Comp Pathol. (2015) 152:9–21. 10.1016/j.jcpa.2014.09.003 25443146

[15] OIE. Chapter 2.8.1. African swine fever. In: Manual of Diagnostic Tests and Vaccines for Terrestrial Animals (Mammals, Birds and Bees). Paris: World Organisation for Animal Health (2012).

[16] Zhao D Liu R Zhang X Li F Wang J Zhang J. Replication and virulence in pigs of the first African swine fever virus isolated in China. Emerg Microbes Infect. (2019) 8:438–47. 10.1080/22221751.2019.1590128 30898043 PMC6455124

[17] Wen X He X Zhang X Zhang X Liu L Guan Y. Genome sequences derived from pig and dried blood pig feed samples provide important insights into the transmission of Africa swine fever virus in China in 2018. Emerg Microbes Infect. (2019) 8:303–6. 10.1080/22221751.2019.1565915 30866784 PMC6455166

[18] Gallardo C Soler A Nurmoja I Cano-Gómez C Cvetkova S Frant M. Dynamics of African swine fever virus (ASFV) infection in domestic pigs infected with virulent, moderate virulent and attenuated genotype II ASFV European isolates. Transbound Emerg Dis. (2021) 68:2826–41. 10.1111/tbed.14222 34273247

[19] Weldu T Lulu W Ghebremedhin T Yibrah T Zhenjiang Z Jiwen Z. Characterization of anti-p54 monoclonal antibodies and their potential use for African swine fever virus diagnosis. Pathogens. (2021) 10:178. 10.3390/pathogens10020178 33562314 PMC7915713

[20] Gómez-Puertas P Rodríguez F Oviedo J Brun A Alonso C Escribano J. The African swine fever virus proteins p54 and p30 are involved in two distinct steps of virus attachment and both contribute to the antibody-mediated protective immune response. Virology. (1998) 243:461–71. 10.1006/viro.1998.9068 9568043

[21] Rodríguez JM García-Escudero R Salas ML Andrés G. African swine fever virus structural protein p54 is essential for the recruitment of envelope precursors to assembly sites. J Virol. (2004) 78:4299–313. 10.1128/JVI.78.8.4299-4313.2004 15047843 PMC374266

[22] García-Mayoral MF Rodríguez-Crespo I Bruix M. Structural models of DYNLL1 with interacting partners: African swine fever virus protein p54 and postsynaptic scaffolding protein gephyrin. FEBS Lett. (2011) 585:53–7. 10.1016/j.febslet.2010.11.027 21094642

[23] Perez-Filgueira DM Camacho FG Gallardo C Resino-Talavan P Blanco E Gomez-Casado E. Optimization and validation of recombinant serological tests for African swine fever diagnosis based on detection of the p30 protein produced in Trichoplusia ni larvae. J Clin Microbiol. (2006) 44:3114–21. 10.1128/JCM.00406-06 16954235 PMC1594705

[24] Fangfeng Y Vlad P Luis G Jeffrey J Raymond R Ying F. Development of a blocking enzyme-linked immunosorbent assay for detection of antibodies against African swine fever virus. Pathogens. (2021) 10:760. 10.3390/pathogens10060760 34204199 PMC8234086

[25] Cubillos C Gómez-Sebastian S Moreno N Nuñez MC Mulumba-Mfumu LK Quembo CJ. African swine fever virus serodiagnosis: a general review with a focus on the analyses of African serum samples. Virus Res. (2013) 173:159–67. 10.1016/j.virusres.2012.10.021 23131491

[26] Gallardo C Reis AL Kalema-Zikusoka G Malta J Soler A Blanco E. Recombinant antigen targets for serodiagnosis of African swine fever. Clin Vaccine Immunol. (2009) 16:1012–20. 10.1128/CVI.00408-08 19420186 PMC2708404

[27] Reis AL Parkhouse RME Penedos AR Martins C Leitão AB. Systematic analysis of longitudinal serological responses of pigs infected experimentally with African swine fever virus. J Gen Virol. (2007) 88:2426–34. 10.1099/vir.0.82857-0 17698651

[28] Ning J Yunwen O Zygmunt P Yongguang Z Jie Z. Roles of African swine fever virus structural proteins in viral infection. J Vet Res. (2017) 61:135–43. 10.1515/jvetres-2017-0017 29978065 PMC5894393

[29] Pamela L Haru T Dirk W Linda D Dave C. Correlation of cell surface marker expression with African swine fever virus infection. Vet Microbiol. (2014) 168:413–19. 10.1016/j.vetmic.2013.12.001 24398227 PMC3969584

[30] Barderas MG Rodríguez F Gómez-Puertas P Avilés M Beitia F Alonso C. Antigenic and immunogenic properties of a chimera of two immunodominant African swine fever virus proteins. Arch Virol. (2001) 146:1681–91. 10.1007/s007050170056 11699955

[31] Ann-Maree C Tatiana AS David AJ Philip GB Rohan TB. An efficient system for high-level expression and easy purification of authentic recombinant proteins. Prot Sci. (2004) 13:1331–9. 10.1110/ps.04618904 15096636 PMC2286746

[32] Mallory EH Maria VM Ping W Andre DL Wei J Raymond RR. Linear epitopes in African swine fever virus p72 recognized by monoclonal antibodies prepared against baculovirus-expressed antigen. J Vet Diagn Invest. (2018) 30:104063871775396. 10.1177/1040638717753966 29327672 PMC6505808

[33] Ying F Andrew P Lia H Eric AN Raymond RR. Production and characterization of monoclonal antibodies against the nucleocapsid protein of SARS-COV. Adv Exp Med Biol. (2006) 581:153–6. 10.1007/978-0-387-33012-9_27 17037523 PMC7139450

[34] Yanhua L Ali T Eric JS Ying F. Identification of porcine reproductive and respiratory syndrome virus ORF1a-encoded non-structural proteins in virus-infected cells. J Gen Virol. (2012) 93:829–39. 10.1099/vir.0.039289-0 22258855

[35] Wang L Mi S Gong W Shi J Madera R Ganges L. A neutralizing monoclonal antibody-based competitive ELISA for classical swine fever C-strain post-vaccination monitoring. BMC Vet Res. (2020) 16:14. 10.1186/s12917-020-2237-6 31937302 PMC6958719

[36] Atuhaire DK Afayoa M Ochwo S Mwesigwa S Ojok L. Prevalence of African swine fever virus in apparently healthy domestic pigs in Uganda. BMC Vet Res. (2013) 9:263. 10.1186/1746-6148-9-263 24369729 PMC3877968

[37] Patrick BN Machuka EM Githae D Banswe G Amimo JO Ongus JR. Evidence for the presence of African swine fever virus in apparently healthy pigs in South-Kivu Province of the Democratic Republic of Congo. Vet Microbiol. (2020) 240:108521. 10.1016/j.vetmic.2019.108521 31902515 PMC7045278

[38] Franzoni G Giudici SD Loi F Sanna D Floris M Fiori M. African swine fever circulation among free-ranging pigs in Sardinia: data from the eradication program. Vaccines. (2020) 8:549. 10.3390/vaccines8030549 32967098 PMC7563918

[39] Sandvik T. Laboratory diagnostic investigations for bovine viral diarrhoea virus infections in cattle. Vet Microbiol. (1999) 64:123. 10.1016/S0378-1135(98)00264-8 10028167

[40] Henriques AM Fagulha T Barros SC Ramos F Duarte M Luís T. Development and validation of a blocking ELISA test for the detection of avian influenza antibodies in poultry species. J Virol Methods. (2016) 236:47–53. 10.1016/j.jviromet.2016.07.006 27421625

[41] Isa G Pfister K Kaaden OR Czerny CP. Development of a monoclonal blocking ELISA for the detection of antibodies against Fowlpox virus. J Vet Med B Infect Dis Vet Public Health. (2002) 49:21–3. 10.1046/j.1439-0450.2002.00533.x 11911587

[42] Castro C Gourley M. Diagnostic testing and interpretation of tests for autoimmunity. J Allergy Clin Immunol. (2010) 125:S238–47. 10.1016/j.jaci.2009.09.041 20061009 PMC2832720

[43] Krasowski MD. Educational case: hemolysis and lipemia interference with laboratory testing. Acad Pathol. (2019) 6:6. 10.1177/2374289519888754 31803827 PMC6876161

[44] Malmquist WA. Serologic and immunologic studies with African swine fever virus. Am J Vet Res. (1963) 24:450–9. 13932609

[45] Sánchez-Vizcaíno JM La Dd Omada A Avilés MM. Editorial: African swine fever. Front Vet Sci. (2021) 7:632292. 10.3389/fvets.2020.632292 33585613 PMC7873590

